# Combined impact of alcohol consumption and metabolic syndrome on liver dysfunction in an elderly Chinese population

**DOI:** 10.1186/s13098-024-01312-0

**Published:** 2024-03-23

**Authors:** Yanrong Zhao, Xiaoxue Yuan, Tianxiang Lin, Qing Yang, Xuewen Jiang, Song Yang, Yinwei Qiu

**Affiliations:** 1 Zhejiang Provincial Center for Disease Control and Prevention, Hangzhou, 310051 China; 2 Beijing Key Laboratory of Emerging Infectious Diseases, Institute of Infectious Diseases, Beijing Ditan Hospital, Capital Medical University, Beijing, 100015 China; 3 Center of Liver Diseases Division 3, Beijing Ditan Hospital, Capital Medical University, Beijing, 100015 China; 4 Beijing Institute of Infectious Diseases, Beijing, 100015 China; 5 National Center for Infectious Diseases, Beijing Ditan Hospital, Capital Medical University, Beijing, 100015 China; 6 National Key Laboratory of Intelligent Tracking and Forecasting for Infectious Diseases, Beijing, 100015 China

**Keywords:** Alcohol consumption, Metabolic disorder, Liver dysfunction

## Abstract

Alcohol consumption and metabolic syndrome(MetS), both prevalent in the general population, frequently co-occur. They are recognized as significant contributors to liver dysfunction, yet their combined effect is often challenging to delineate. This study delves into the compounding influence of alcohol consumption and metabolic disorder on liver dysfunction within an elderly demographic in Zhejiang Province, China. Our findings spotlight a heightened risk of liver dysfunction among females, younger individuals, rural dwellers, those with minimal educational attainment, single individuals, and those diagnosed with MetS. We also discerned a positive correlation correlation between the number of MetS components and the propensity for liver dysfunction. Furthermore, the risk of liver dysfunction escalated in tandem with the frequency of alcohol consumption. Interestingly, a prolonged abstinence period (≥ 5 years) seemed to mitigate this risk. Our research underscores the significance of refraining from excessive alcohol consumption, embracing a healthy lifestyle, and managing MetS components-especially triglyceride levels-for effective prevention of liver dysfunction.

## Introduction

Liver dysfunction poses a critical public health issue, with its pathogenesis and progression influenced by a myriad of factors. Significantly, alcohol consumption and metabolic syndrome(MetS) have been identified as key contributors. These conditions exhibit a high prevalence within the general population and frequently coexist, creating a complex web of health challenges [1]. They are linked to a wide array of health complications, encompassing chronic liver disease, hepatocellular carcinoma (HCC), and other liver-related outcomes such as hepatic decompensation or the need for liver transplantation [2, 3]. Intriguingly, metabolic disorder and alcohol not only independently instigate liver disease but also act synergistically to accelerate its progression. This intricate interplay underscores the importance of comprehensive understanding and strategic management of these variables in mitigating liver disease [4].

Epidemiological and experimental evidence strongly suggest that alcohol and metabolic disorder have additive or synergistic effects in the development and progression of liver disease [1, 5]. Biopsy-based studies have found that, compared to individuals with normal weight, the incidence of fatty degeneration, inflammation, extensive fibrosis, or cirrhosis is higher in obese patients who are heavy drinkers [6]. The harmful interaction between these two conditions appears to be not only additive but also multiplicative [7]. In patients with over-consumption of alcohol and obesity or MetS, the primary driver of disease progression may be alcohol, with metabolic factors serving as modulators of the disease [8]. Furthermore, the combination of MetS and excessive alcohol consumption may synergistically increase the risk of HCC. Therefore, the presence of obesity and T2DM are risk factors for HCC in ALD patients [9], while excessive alcohol consumption increases the risk of HCC in patients with MetS [10].

As far as we know, the relationship between alcohol consumption, metabolic disorder and liver dysfunction remains under-characterized. So far, no study has investigated the combined impact of alcohol consumption and metabolic disorder on liver dysfunction in the Asian population, particularly among older Chinese individuals. This study has two main objectives. First, to investigate the individual associations between alcohol consumption, metabolic disorder, and liver dysfunction among older Chinese individuals. Second, to provide a comprehensive characterization of alcohol consumption and metabolic disorder on liver dysfunction.

## Methods

### Study population

Between January 1, 2022 and December 31, 2022, a total of 5,697,488 older adults (≥ 65 years old) participated in a physical examination organized by the community health centers in Zhejiang Province. Interested sociodemographic data, liver function indicators, information on MetS, and self-reported alcohol consumption history were extracted from the Zhejiang provincial electronic health record (EHR) system using a standardized data extraction form.

Variables associated with sociodemographic characteristics included gender, birthdate, residence, education and marital status. Liver function indicators included alanine aminotransferase (ALT) and aspartate aminotransferase (AST). Variables related to MetS information included body mass index (BMI), waist circumference (WC), systolic blood pressure (SBP), diastolic blood pressure (DBP), fasting blood glucose (FBG), triglyceride (TG), and high-density lipoprotein cholesterol (HDL-C). The self-reported history of alcohol intake consisted of drinking status (former or current drinkers), drinking frequency (occasionally, usually or daily), and years of alcohol abstinence.

**Fig. 1:**
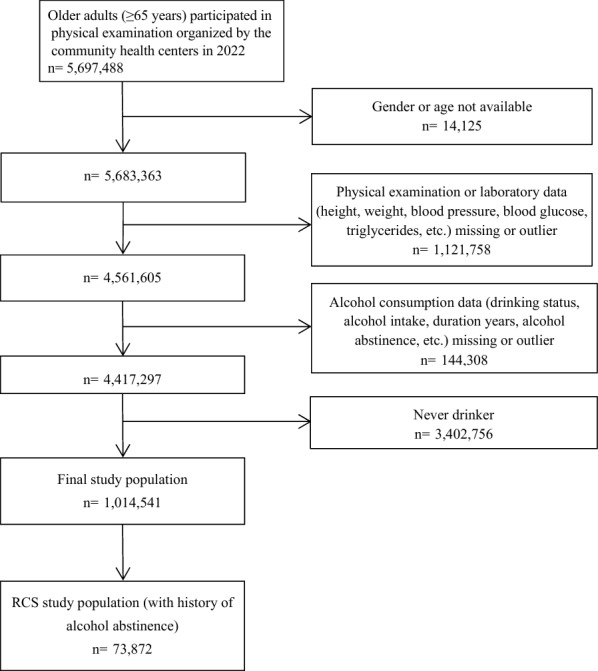
Flowchart of study population selection

The final data analysis included 1,014,541 subjects with a history of alcohol consumption and 73,872 with a history of alcohol abstinence for the restricted cubic splines (RCS) study on nonlinear relationships between years of alcohol abstinence and liver dysfunction. Detailed information on participant selection is shown in Fig. 1.

### Study definitions

ALT and AST are the two most important indicators of liver function. According to local laboratory criteria and literature references, liver dysfunction were defined as ALT level greater than 40 U/L or AST level greater than 37 U/L in men, and ALT or AST level greater than 31 U/L in women [11–14].

MetS was required at least one out of five metabolic components for diagnosis [15, 16]. Based on the special body types and health conditions of the Chinese population, the definition of MetS and the cutoff criteria include:BMI ≥ 24 or WC ≥ 90 cm for men and ≥ 85 cm for women; FBG ≥ 5.6 mmol/L; SBP/DBP ≥ 140/90 mmHg; TG ≥ 1.7 mmol/L; HDL-C ≤ 1.0 mmol/L for men and ≤ 1.3 mmol/L for women [17, 18].

### Statistical analysis

The populations were divided into two groups based on the presence or absence of liver dysfunction. The proportion was calculated for categorical variables in each category. For non-normally distributed continuous variables, we used the median with interquartile range (IQR) to summarize the data. While for normally distributed continuous variables, we used the mean ± standard deviation. We assessed the normality of continuous variables using QQ plots and histograms. To compare the differences in sociodemographic characteristics and potential risk factors between the subjects, we used the χ2 test and Kruskal–Wallis rank sum test for categorical and continuous variables, respectively.

Binary logistic regression analysis was conducted to examine factors associated with liver dysfunction. In the multivariate regression models, we applied a backward stepwise selection strategy. Prior to performing the multivariate analysis, we conducted univariate analysis for each factor to determine if the covariance inclusion criteria were met and to identify variables that affect liver function. Among subjects with a history of alcohol consumption, the effects of potential interaction between alcohol consumption and MetS on liver dysfunction were assessed on multiplicative scale by including cross-product terms in the logistic multivariate model and additive scale with the relative excess risk index (RERI), attributable proportion (AP), synergy index (S).

Subjects with a history of alcohol abstinence were selected to analyze the potential nonlinear relationships between years of alcohol abstinence and liver dysfunction. A logistic regression model with RCS [19, 20] was conducted with 4 knots at the 5th, 35th, 65th, and 90th centiles to flexibly model the association. The reference value was set at the 50th centiles (5 years). The RCS model was adjusted for age, gender, residence, education, marital status, and presence of five metabolic components. As the associations of alcohol abstinence years and liver dysfunction were approximately log linear below and above their medians, we additionally used a segmented logistic regression to calculate ORs in different parts of the curve.

All the analyses were performed in R version 4.3.1. P value < 0.05 (two-sided) was considered statistically significant.

## Results

### Sociodemographic characteristics

Of the 1,014,541 participants with a history of alcohol consumption, 847,049 (83.49%) were men. The median (IQR) age was 71 (68–75) years. A total of 940,669 (92.72%) participants reported current alcohol consumption, while 73872 (7.28%) were abstainers. 246,784 (24.32%) reported occasionally drinking, and 693,885 (68.39%) reported usually or daily drinking. Additionally, 35,819 (3.53%) reported abstaining from alcohol for less than 5 years, and 38,053 (3.75%) reported abstaining for 5 years or more.

All the participants were grouped into normal and abnormal liver function subgroups. A significant difference was detected between the 2 subgroups in terms of gender, age, residence, education level composition, marital and drinking status. The comparison results of baseline data between normal and abnormal liver function subgroups were summarized in the Table 1. Participants with liver dysfunction (145033, 14.30%) were generally younger and had a higher prevalence in women. They also tended to live in rural areas, have lower levels of education, and be single.

**Table 1:** The sociodemographics characteristics of older adults with different states of liver function

Characteristic	Overall, N = 1,014,541^1^	Normal, N = 869,508^1^	Abnormal, N = 145,033^1^	p-value^2^
Gender				< 0.001
Male	847,049 (83.49%)	731,323 (84.11%)	115,726 (79.79%)	
Female	167,492 (16.51%)	138,185 (15.89%)	29,307 (20.21%)	
Age groups, years				< 0.001
65–69	407,846 (40.20%)	345,174 (39.70%)	62,672 (43.21%)	
70–74	322,689 (31.81%)	275,872 (31.73%)	46,817 (32.28%)	
75–79	173,102 (17.06%)	150,102 (17.26%)	23,000 (15.86%)	
≥ 80	110,904 (10.93%)	98,360 (11.31%)	12,544 (8.65%)	
Residence				0.003
Urban	471,969 (46.52%)	405,015 (46.58%)	66,954 (46.16%)	
Rural	542,572 (53.48%)	464,493 (53.42%)	78,079 (53.84%)	
Education				< 0.001
Primary school or no school	648,455 (63.92%)	553,150 (63.62%)	95,305 (65.71%)	
Secondary school	223,850 (22.06%)	192,782 (22.17%)	31,068 (21.42%)	
College and above	13,437 (1.32%)	11,774 (1.35%)	1,663 (1.15%)	
Unknown	128,799 (12.70%)	111,802 (12.86%)	16,997 (11.72%)	
Marital status				< 0.001
Single^#^	82,225 (8.10%)	69,730 (8.02%)	12,495 (8.62%)	
Married	841,680 (82.96%)	721,099 (82.93%)	120,581 (83.14%)	
Unknown	90,636 (8.93%)	78,679 (9.05%)	11,957 (8.24%)	
Drinking status				< 0.001
Occasionally	246,784 (24.32%)	214,828 (24.71%)	31,956 (22.03%)	
Usually/Daily	693,885 (68.39%)	589,948 (67.85%)	103,937 (71.66%)	
Alcohol abstinence years < 5	35,819 (3.53%)	31,003 (3.57%)	4,816 (3.32%)	
Alcohol abstinence years ≥ 5	38,053 (3.75%)	33,729 (3.88%)	4,324 (2.98%)	

^1^n (%)

^2^Pearson's Chi-squared test

^#^Single: unmarried, divorced or widowed

### Liver dysfunction and the metabolic syndrome

The median (IQR) ALT was 17.00 (14.00, 22.00) in the normal liver function group and 39.00 (28.00, 50.00) in the abnormal group. The median (IQR) AST was 24.00 (20.00, 28.00) in the normal liver function group and 42.00 (38.00, 51.00) in the abnormal group. Compared to the group with normal liver function, participants with abnormal liver function exhibited elevated levels of BMI, WC, SBP, DBP, FBG, TG, HDL-C and a greater prevalence of five MetS, as well as a higher number of MetS (Table 2).

**Table 2:** The sex-specific prevalence of MetSs of older adults with different states of liver function

Characteristic	all			Male			Female			p-value^2^
	Overall, N = 1,014,541^1^	Normal, N = 869,508^1^	Abnormal, N = 145,033^1^	Overall, N = 847,049^1^	normal, N = 731,323^1^	abnormal, N = 115,726^1^	Overall, N = 167,492^1^	normal, N = 138,185^1^	abnormal, N = 29,307^1^	
BMI	23.63 (3.12)	23.58 (3.05)	23.93 (3.48)	23.59 (3.07)	23.55 (3.01)	23.81 (3.42)	23.84 (3.36)	23.72 (3.29)	24.40 (3.65)	< 0.001
WC	84.60 (9.04)	84.45 (8.90)	85.54 (9.80)	84.84 (8.98)	84.71 (8.84)	85.72 (9.78)	83.38 (9.23)	83.08 (9.07)	84.84 (9.85)	< 0.001
SBP	139.32 (17.90)	139.14 (17.87)	140.37 (18.05)	139.04 (17.80)	138.87 (17.76)	140.15 (17.99)	140.70 (18.34)	140.59 (18.35)	141.22 (18.26)	< 0.001
DBP	80.36 (10.12)	80.25 (10.08)	81.04 (10.31)	80.59 (10.12)	80.46 (10.08)	81.35 (10.33)	79.24 (10.04)	79.12 (10.02)	79.79 (10.11)	< 0.001
FBG	5.60 (5.00, 6.20)	5.50 (5.00, 6.10)	5.70 (5.00, 6.58)	5.51 (5.00, 6.20)	5.50 (5.00, 6.10)	5.70 (5.00, 6.50)	5.70 (5.02, 6.20)	5.60 (5.00, 6.15)	5.90 (5.10, 6.70)	< 0.001
TG	1.30 (0.98, 2.00)	1.30 (0.96, 2.00)	1.54 (1.00, 2.21)	1.28 (0.93, 2.00)	1.24 (0.91, 1.99)	1.50 (1.00, 2.22)	1.54 (1.02, 2.00)	1.50 (1.00, 2.00)	1.70 (1.10, 2.20)	< 0.001
HDL-C	1.49 (1.20, 1.93)	1.49 (1.20, 1.91)	1.53 (1.21, 2.00)	1.47 (1.19, 1.89)	1.46 (1.19, 1.87)	1.51 (1.20, 2.00)	1.60 (1.29, 2.00)	1.60 (1.30, 2.00)	1.59 (1.27, 2.00)	< 0.001
BMI ≥ 24 or WC ≥ 80 cm(M) 85(F)	490,906 (48.39%)	414,519 (47.67%)	76,387 (52.67%)	398,784 (47.08%)	340,522 (46.56%)	58,262 (50.34%)	92,122 (55%)	73,997 (53.55%)	18,125 (61.85%)	< 0.001
BP ≥ 140/90 mmHg	553,412 (54.55%)	470,383 (54.10%)	83,029 (57.25%)	457,753 (54.04%)	391,850 (53.58%)	65,903 (56.95%)	95,659 (57.11%)	78,533 (56.83%)	17,126 (58.44%)	< 0.001
FBG ≥ 5.6 mmol/L	507,746 (50.05%)	427,483 (49.16%)	80,263 (55.34%)	417,524 (49.29%)	354,713 (48.50%)	62,811 (54.28%)	90,222 (53.87%)	72,770 (52.66%)	17,452 (59.55%)	< 0.001
TG ≥ 1.70 mmol/L	361,262 (35.61%)	295,266 (33.96%)	65,996 (45.50%)	286,355 (33.81%)	235,016 (32.14%)	51,339 (44.36%)	74,907 (44.72%)	60,250 (43.60%)	14,657 (50.01%)	< 0.001
HDL-C ≤ 1.0(M) 1.3(F)	139,055 (13.71%)	117,113 (13.47%)	21,942 (15.13%)	94,873 (11.20%)	81,076 (11.09%)	13,797 (11.92%)	44,182 (26.38%)	36,037 (26.08%)	8,145 (27.79%)	< 0.001
Number of Metabolic Syndromes										< 0.001
0	116,286 (11.46%)	103,348 (11.89%)	12,938 (8.92%)	104,864 (12.38%)	93,518 (12.79%)	11,346 (9.80%)	11,422 (6.82%)	9,830 (7.11%)	1,592 (5.43%)	
1	252,950 (24.93%)	222,364 (25.57%)	30,586 (21.09%)	220,762 (26.06%)	194,881 (26.65%)	25,881 (22.36%)	32,188 (19.22%)	27,483 (19.89%)	4,705 (16.05%)	
2	288,990 (28.48%)	250,442 (28.80%)	38,548 (26.58%)	242,423 (28.62%)	211,253 (28.89%)	31,170 (26.93%)	46,567 (27.80%)	39,189 (28.36%)	7,378 (25.17%)	
3	225,120 (22.19%)	188,560 (21.69%)	36,560 (25.21%)	180,958 (21.36%)	152,601 (20.87%)	28,357 (24.50%)	44,162 (26.37%)	35,959 (26.02%)	8,203 (27.99%)	
4	109,884 (10.83%)	88,134 (10.14%)	21,750 (15.00%)	83,403 (9.85%)	67,363 (9.21%)	16,040 (13.86%)	26,481 (15.81%)	20,771 (15.03%)	5,710 (19.48%)	
5	21,311 (2.10%)	16,660 (1.92%)	4,651 (3.21%)	14,639 (1.73%)	11,707 (1.60%)	2,932 (2.53%)	6,672 (3.98%)	4,953 (3.58%)	1,719 (5.87%)	

BMI = body mass index; WC = waist circumference; SBP = systolic blood pressure; DBP = diastolic blood pressure; FBG = fasting blood glucose;TG = triglycerides; HDL-C = high-density lipoprotein cholesterol

^1^Mean (SD); Median (IQR); n (%)

^2^Pearson's Chi-squared test; Kruskal–Wallis rank sum test

### The association between alcohol consumption, metabolic syndrome, and liver dysfunction

The unadjusted ORs for liver dysfunction were significantly higher for female participants, younger individuals, those living in rural areas, those with lower education levels, and those who were single, along with MetS. Individuals with any MetS were all at a higher risk. There was a clear positive correlation relationship between the numbers of MetS and the risk of liver dysfunction.

After adjusting for other covariates in the multivariate analysis, the association between alcohol consumption and the risk of liver dysfunction was significant. Higher frequency of drinking posed a higher risk compared to occasional drinkers (adjusted OR 1.26; 95% CI 1.24–1.27). The risk of liver dysfunction remained higher for individuals abstaining from alcohol for less than 5 years compared to occasional drinkers (adjusted OR 1.12; 95% CI 1.08–1.16). However, extended abstinence (≥ 5 years) resulted in a lower risk (adjusted OR 0.94; 95% CI 0.91–0.98). The decrease in HDL-C showed a weak but positive protective effect on liver function (adjusted OR 0.98; 95% CI 0.97–1.00), while the presence of other MetS remained risk factors, particularly higher TG levels (adjusted OR 1.52; 95% CI 1.51–1.54). It is noteworthy that the number of MetS was not included in the multivariate model (Table 3).

**Table 3:** Logistic regression analyses of influence and risk factors for liver dysfunction

Variables	Univariate Regression Analysis			Multivariate Regression Analysis^2^		
	*OR*^1^	95% *CI*^1^	*p*-value	*OR*^1^	95% *CI*^1^	*p*-value
Gender						
Male	–	–		–	–	
Female	1.34	1.33, 1.36	< 0.001	1.31	1.29, 1.33	< 0.001
Age groups, years						
65–69	–	–		–	–	
70–74	0.93	0.92, 0.95	< 0.001	0.94	0.93, 0.95	< 0.001
75–79	0.84	0.83, 0.86	< 0.001	0.86	0.85, 0.88	< 0.001
≥ 80	0.70	0.69, 0.72	< 0.001	0.74	0.72, 0.75	< 0.001
Residence						
Urban	–	–		–	–	
Rural	1.02	1.01, 1.03	0.003	1.02	1.01, 1.04	< 0.001
Education						
Primary school or no school	–	–		–	–	
Secondary school	0.94	0.92, 0.95	< 0.001	0.93	0.92, 0.94	< 0.001
College and above	0.82	0.78, 0.86	< 0.001	0.87	0.83, 0.92	< 0.001
Unknown	0.88	0.87, 0.90	< 0.001	0.82	0.80, 0.84	< 0.001
Marital status						
Single^#^	–	–		–	–	
Married	0.93	0.91, 0.95	< 0.001	0.97	0.95, 0.99	0.011
Unknown	0.85	0.83, 0.87	< 0.001	1.06	1.03, 1.10	< 0.001
Alcohol use						
Occasionally	–	–		–	–	
Sometimes/Daily	1.18	1.17, 1.20	< 0.001	1.26	1.24, 1.27	< 0.001
Alcohol abstinence years < 5	1.03	1.00, 1.07	0.070	1.12	1.08, 1.16	< 0.001
Alcohol abstinence years ≥ 5	0.86	0.83, 0.89	< 0.001	0.94	0.91, 0.98	0.001
BMI ≥ 24 or WC ≥ 80 cm(M)85(F)						
No	–	–		–	–	
Yes	1.22	1.21, 1.24	< 0.001	1.08	1.07, 1.10	< 0.001
BP ≥ 140/90 mmHg						
No	–	–		–	–	
Yes	1.14	1.12, 1.15	< 0.001	1.09	1.08, 1.10	< 0.001
FBG ≥ 5.6 mmol/L						
No	1.28	1.27, 1.30	< 0.001	–	–	
Yes	1.28	1.27, 1.30	< 0.001	1.19	1.17, 1.20	< 0.001
TG ≥ 1.70 mmol/L						
No	–	–		–	–	
Yes	1.62	1.61, 1.64	< 0.001	1.52	1.51, 1.54	< 0.001
HDL-C ≤ 1.0(M) 1.3(F)						
No	–	–		–	–	
Yes	1.15	1.13, 1.16	< 0.001	0.98	0.97, 1.00	0.042
Number of MetSs						
0	–	–				
1	1.10	1.08, 1.12	< 0.001			
2	1.23	1.20, 1.26	< 0.001			
3	1.55	1.52, 1.58	< 0.001			
4	1.97	1.93, 2.02	< 0.001			
5	2.23	2.15, 2.31	< 0.001			

BMI = body mass index; WC = waist circumference; SBP = systolic blood pressure; DBP = diastolic blood pressure; FBG = fasting blood glucose;TG = triglycerides; HDL-C = high-density lipoprotein cholesterol

^#^Single: unmarried, divorced or widowed

^1^OR = Odds Ratio, CI = Confidence Interval

^2^ “Number of MetSs” was not included in the multivariate model

### The interaction effect between alcohol consumption and metabolic syndrome on liver dysfunction

To further investigate the potential interaction effect between alcohol consumption and MetS on liver dysfunction, alcohol consumption and MetS were divided into two categories (drink occasionally vs. drink usually/daily; and no MetS vs. MetS). The baseline information and interaction effects were summarized in Table 4.

**Table 4:** Logistic regression analysis of the interactive items between alcohol consumption and MetS on liver dysfunction

Variables	n(presence of dysfunction /absence of dysfunction)	OR(95% CI)	OR-Int ^a^	RERI^b^	AP^b^	S^b^
Drink occasionally + no MetS	2361/23323	Ref	0.90 (0.86, 0.95)	-0.02 (-0.08, 0.03)	-0.01 (-0.04, 0.02)	0.97 (0.91, 1.04)
Drink usually/daily + no MetS	9771/72491	1.37 (1.31, 1.44)				
Drink occasionally + MetS	29595/191505	1.48 (1.41, 1.54)				
Drink usually/daily + MetS	94166/517457	1.82 (1.74, 1.90)				

Model is adjusted for gender, age group, residence area, education level, and marital status; MetS = metabolic syndrome;

^a^ OR- int is assessed on the multiplicative scale by including cross-product terms in the model;

^b^ RERI, AP, and S are assessed on the additive scale. * p < 0.05

The logistic regression analysis revealed that after adjusting for confounders such as gender, age group, residence area, education level, and marital status, a significant sub-multiplicative interaction (0.90; 95% CI: 0.86, 0.95) was observed between alcohol consumption and MetS on liver dysfunction. However, the confidence intervals (CIs) of interactive indexes RERI (− 0.02; 95% CI − 0.08, 0.03) and AP (− 0.01; 95% CI − 0.04, 0.02) included 0, and 1 was involved in the CIs of S (0.97; 95% CI 0.91, 1.04), suggesting that there was not an additive interaction.

### The association between years of alcohol abstinence and liver dysfunction

**Fig. 2:**
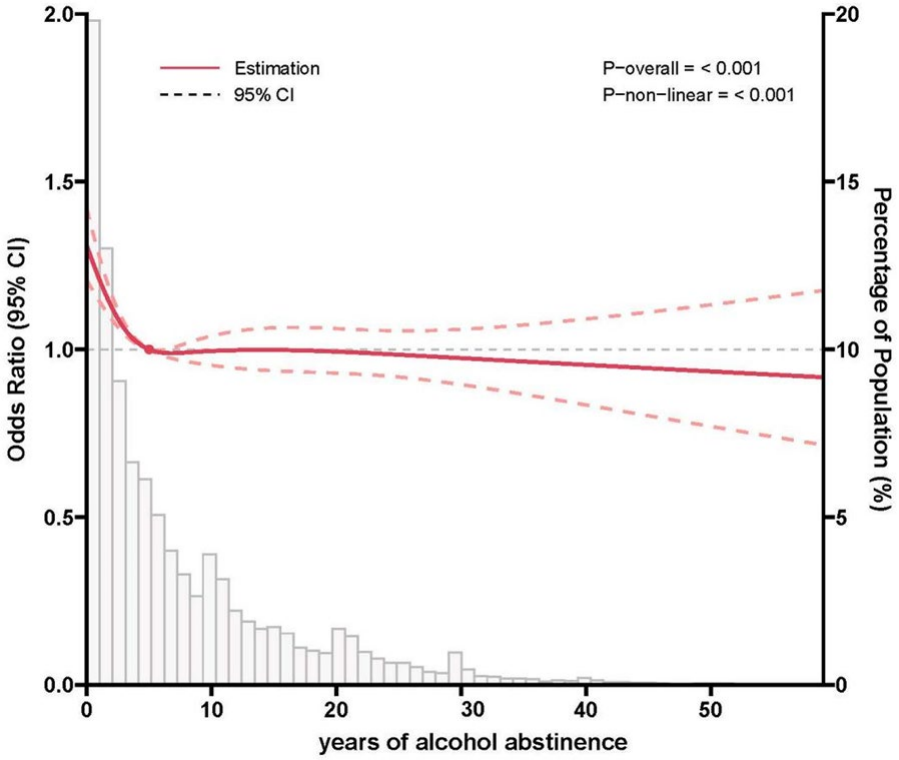
Association between years of alcohol abstinence and liver function using a Restricted Cubic Spline Regression Model. Results were adjusted for age, gender, residence, education, marital status, and presence of five metabolic diseases

The median (IQR) duration of alcohol abstinence years was 5 (2–11) years. After adjusting for potential confounders, the effect of alcohol abstinence years on liver dysfunction fitted a non-linear spline model (P < 0.001), restricted cubic splines showed an L-shaped curve (Fig. 2). The adjusted OR for alcohol abstinence duration less than 5 years was 0.946 (95% CI 0.922–0.970). However, when duration of alcohol abstinence exceeded 5 years, the risk remained relatively constant, with an adjusted OR 1.000 (95% CI 0.996–1.004).

## Discussion

This study aimed to investigate the relationship among alcohol consumption, metabolic disorder, and liver dysfunction in the elderly population of Zhejiang province, China. To our knowledge, this is the first study to comprehensively assess the combined impact of alcohol consumption and metabolic disorder on liver dysfunction in an Asian population, particularly among Chinese elderly individuals. The current study suggests that the risk of liver dysfunction is associated with numerous factors, including gender, age, residence, education level, marital status, alcohol consumption behavior, and the presence of MetS. Our findings underscore the importance of avoiding excessive alcohol consumption, maintaining a healthy lifestyle, and effectively controlling various components of MetS, especially triglyceride levels, for the prevention of liver dysfunction. In addition, our research provides a comprehensive perspective on the impact of alcohol consumption and metabolic disorder on liver dysfunction.

MetS has been identified as an independent driver of cirrhosis and liver-related diseases. In a recent US study, MetS was the largest contributor to population-level HCC (attributable fraction: 32%) [21]. Additionally, the presence of MetS can predict liver-related mortality in various chronic liver diseases [22]. A recent large study in the US involving 271,906 NAFLD patients and an average 9-year follow-up reported that each additional metabolic feature (diabetes, obesity, hypertension, dyslipidemia) was associated with a stepwise increase in the risk of liver-related outcomes (cirrhosis or HCC). All four metabolic features independently influenced the risk, but diabetes had the strongest correlation with HCC events (hazard ratio [HR]: 2.8) [23]. Metabolic disorder, particularly MetS, is also considered a significant factor leading to liver dysfunction [24]. MetS includes characteristics such as obesity, hypertension, hyperglycemia, elevated triglyceride levels, and low high-density lipoprotein cholesterol levels, all of which have been proven to be associated with the progression of liver disease [25]. Compared to the group with normal liver function, participants in the group with abnormal liver function demonstrated adverse performances on multiple metabolic indicators. They exhibited significantly elevated levels of BMI, WC, SBP, DBP, FBG, TG, and HDL-C. Furthermore, participants in the group with abnormal liver function also had a higher prevalence and quantity of MetS. These results suggest a clear positive correlation relationship between the number of MetS and the risk of liver dysfunction, i.e., the more MetS, the higher the risk of liver dysfunction. Abnormal liver function may be a significant component of MetS, or one of the outcomes of MetS. Therefore, improving the management of MetS, such as weight loss, dietary improvement, and increased physical activity, may help in the prevention and management of liver dysfunction. Additionally, high-density lipoprotein cholesterol (HDL-C) is a beneficial lipid with antioxidative, anti-inflammatory, and anticoagulant effects [26]. A reduction in HDL-C levels may be associated with metabolic abnormalities and the occurrence of liver dysfunction [27]. Thus, improving lipid metabolism, especially by increasing HDL-C levels, may help protect liver function. Therefore, the higher level of HDL-C found in patients with liver dysfunction in this study may be a feedback mechanism. Interestingly, in the univariate analysis, low levels of HDL-C were a risk factor for abnormal liver function, while in the multivariate analysis, low levels of HDL-C exhibited a weak but positive protective effect on liver function. Nevertheless, the presence of other MetS remains still remains a risk factor, especially elevated triglyceride (TG) levels. Our research also found that in populations with MetS, particularly those with higher TG levels, the risk of liver dysfunction significantly increased (adjusted OR 1.52; 95% CI 1.51–1.54). These results suggest that improving lipid metabolism can have a positive impact on liver function. However, while the presence and quantity of MetS seem to be associated with an increased risk of liver dysfunction, we also found that female participants, younger individuals, people living in rural areas, individuals with lower education levels, single individuals, and those with MetS had a significantly increased risk of liver dysfunction. This might be related to lifestyle, socioeconomic status, and differences in access to and understanding of health information in these groups. This further enhances the understanding of the relationship between abnormal liver function and specific populations and metabolic disorders.

It is estimated that fatty degeneration occurs in drinkers who consume 4–5 standard drinks daily. Persistent drinking leads to approximately 25% of individuals developing alcohol-related fatty hepatitis or cirrhosis, eventually progressing to portal hypertension and liver cancer [28, 29]. Most patients remain asymptomatic until late stages of chronic liver disease unless they develop alcohol-related hepatitis [30]. This latter condition is a severe form of ALD characterized by rapid jaundice, discomfort, decompensated liver disease, and coagulation dysfunction, with a high mortality rate (up to 50% at 3 months) [31]. Cross-sectional data from the United States indicate that the co-occurrence of high levels of alcohol consumption and obesity increases the risk of abnormal liver enzyme activity more than either risk factor alone [32]. Recently, these results were confirmed by a data from a prospective population-based cohort, indicating that an average daily alcohol intake of at least 40 g, in conjunction with obesity, is associated with liver enzyme abnormalities seven years later [33]. A longitudinal cohort study involving 52,066 type 2 diabetes patients found that the majority of the liver burden may be attributed to alcohol consumption rather than obesity [34]. Model studies confirmed that excessive alcohol consumption contributes more to liver disease relative to metabolic factors [35]. Alcohol can cause direct toxic damage to the liver, leading to hepatitis, cirrhosis, and even liver cancer [36]. In the multivariate analysis, we corrected for the effects of other covariates and found a significant correlation between alcohol consumption and the risk of liver dysfunction. The higher the frequency of alcohol consumption, the greater the risk of liver dysfunction. This is consistent with previous research findings, where alcohol consumption is one of the important risk factors leading to liver disease. Moreover, our study also shows that the impact of years of abstaining from alcohol on liver dysfunction is non-linear. People who have abstained from alcohol for less than five years are still more likely to have liver dysfunction than those who drink occasionally, but once the abstinence period exceeds five years, the risk remains relatively constant. This may suggest that the liver's ability to self-repair and recover is effective within a certain time frame, but the risk of liver dysfunction does not further decrease after prolonged abstinence, possibly due to other factors such as age, genetics or lifestyle, and the liver's repair ability may weaken over time [37, 38]. This indicates that abstaining from alcohol has a positive impact on liver function recovery and underscores the importance of long-term abstinence.

Alcohol consumption and MetS are very common among the population and often coexist, indicating a complex relationship between alcohol and the components of MetS [4]. Both alcohol consumption and metabolic disorder are considered important factors leading to liver dysfunction, but their synergistic impact is more complex to analyze. Our results indicate that MetS increases the risk of liver-related outcomes regardless of the level of alcohol consumption. Metabolic components appear to alter the positive correlation relationship between alcohol intake and the risk of liver disease. On the other hand, we have divided the sample into distinct categories based on the frequency of alcohol intake (either occasional or usual/daily) and the presence of MetS disorder (either absent or present), aiming to delve into the combined impact of alcohol consumption and metabolic disorder on liver dysfunction. The results of this statistical analysis showed a significant sub-multiplicative interaction between alcohol consumption and metabolic disorder concerning liver dysfunction, without a clear additive interaction, indicating that the observed relationship between alcohol consumption, metabolic disorder, and liver dysfunction does not appear to be additive. Although the multiplicative interaction between alcohol consumption and metabolic disorder is significant and suggests a compounded, albeit less than expected, impact on liver dysfunction, the lack of an additive interaction highlights the nuanced ways in which these factors work together. These results contribute to the understanding of the complex interplay between lifestyle factors and disease, and they reinforce the need for personalized approaches in the prevention and management of liver dysfunction. Considering the risk stratification of both alcohol consumption and metabolic abnormalities may help identify individuals at risk of liver-related outcomes at an early stage. When both alcohol consumption and metabolic disorder are present, they may interact synergistically, accelerating liver damage. For instance, alcohol consumption could exacerbate symptoms of MetS and the syndrome could reduce the liver's tolerance to alcohol, thereby increasing the risk of liver dysfunction [1]. Hence, alcohol consumption and metabolic disorder could create a vicious cycle, aggravating liver damage. Recently, the utilization of a non-invasive liver fibrosis scoring method, FIB-4, has revealed a positive correlation relationship between alcohol intake and the progression of SLD (Steatotic Liver Disease), providing essential insights for the clinical management of SLD [39]. These findings underscore the importance of avoiding excessive alcohol consumption and managing metabolic diseases in maintaining liver health. Intervention strategies targeting these two factors could help reduce the risk of liver dysfunction, including limiting alcohol intake, improving dietary habits, increasing physical activity, and controlling weight. Meanwhile, those with existing alcohol consumption habits or metabolic diseases may require more aggressive and proactive interventions to prevent the occurrence and progression of liver dysfunction.

It's important to note that the study has several limitations. Firstly, it is cross-sectional and cannot establish causality. Further long-term follow-up studies are necessary. Secondly, the study was conducted in one region of China, potentially introducing regional bias. Lastly, this study did not include testing for gamma-GTP. In future research, consideration will be given to including this indicator and to further explore any potential associations with the components of MetS, providing additional valuable information.

## Conclusion

In summary, our study underscores the association between liver dysfunction and specific populations, alcohol consumption, lipid metabolism, and MetS. These findings bear significant clinical and public health implications for the prevention and management of liver dysfunction. Future research should include new prospective studies to better describe the clinical course of patients with metabolic disorder and varying degrees of alcohol consumption, to provide new biomarkers for disease diagnosis and monitoring, and to evaluate the effectiveness of treatment methods for patients with dual-pathogenesis NAFLD and ALD.

## Data Availability

The data used to support the findings of this study are included within the article.

## References

[1] Fredrik A, Christopher DB, Carlos JP, Ville M, Silvia S (2023). Alcohol consumption and MetS: clinical and epidemiological impact on liver disease. J Hepatol.

[2] Laurens AK, Robert JK, Willem PB (2023). Metabolic dysfunction-associated fatty liver disease and excessive alcohol consumption are both independent risk factors for mortality. Hepatology.

[3] Elisa P, Guillem P, Pere T, Jordi G, Emma A, Carmen E (2021). Interaction between MetS and alcohol consumption, risk factors of liver fibrosis: a population-based study. Liver Int.

[4] Mônica RS, Margareth FD, José EM, Maria SA (2012). MetS and risk factors for non-alcoholic fatty liver disease. Arq Gastroenterol.

[5] Zobair MY, Maria S, Janus O, Yusuf Y, Ajay D, Yuichiro E (2019). Effects of alcohol consumption and MetS on mortality in patients with nonalcoholic and alcohol-related fatty liver disease. Clin Gastroenterol Hepatol.

[6] Bruno R, Axel B, David F, Frédérique C, Pierre B, Ct J-C (2002). Risk factors of fibrosis in alcohol-induced liver disease. Hepatology.

[7] Hamish I, Colin JC, Esther A, Tim RC, Victoria H, John D (2022). Characterizing the risk interplay between alcohol intake and body mass index on cirrhosis morbidity. Hepatology.

[8] John BW, Steven M, Suthat L, Sebastian M, Guruprasad PA, Florian E (2021). Obesity, diabetes, coffee, tea, and cannabis use alter risk for alcohol-related cirrhosis in 2 large cohorts of high-risk drinkers. Am J Gastroenterol.

[9] Nathalie GC, Cendrine C, Valérie B, Isabelle A, Jean-Marc P, Frédéric O (2018). Estimate of hepatocellular carcinoma incidence in patients with alcoholic cirrhosis. J Hepatol.

[10] Rohit L, Hwai-I Y, Jun S, David B, Elizabeth BC, Uchenna I (2013). Synergism between obesity and alcohol in increasing the risk of hepatocellular carcinoma: a prospective cohort study. Am J Epidemiol.

[11] Michael L, Danny W, Jordan S, Alexander SV, Stephen FH, Lawrence F (2023). Early liver specialist consultation is associated with faster biochemical resolution of severe immune checkpoint inhibitor-induced hepatitis. J Natl Compr Canc Netw.

[12] Jihye L, Won-Mook C, Ju HS, Danbi L, Kang MK, Young-Suk L (2022). Efficacy and safety of tenofovir alafenamide versus tenofovir disoproxil fumarate in treatment-naïve chronic hepatitis B. Liver Int.

[13] Yang L, Chunyang L, Jian W, Chuanwu Z, Li Z, Fang J (2020). A case series of COVID-19 patients with chronic hepatitis B virus infection. J Med Virol.

[14] Takaomi K, Kento I, Takashi K, Anna O, Michihiro I, Yasushi H (2020). Lubiprostone in patients with non-alcoholic fatty liver disease: a randomised, double-blind, placebo-controlled, phase 2a trial. Lancet Gastroenterol Hepatol.

[15] Fredrik A, Jaana H, Pauli P, Antti J (2017). Binge drinking and the risk of liver events: a population-based cohort study. Liver Int.

[16] Wang L, Peng W, Zhao Z, Zhang M, Shi Z, Song Z (2021). Prevalence and Treatment of Diabetes in China, 2013–2018. JAMA.

[17] The MetS Research Consortium of Chinese Diabetes Society (2004). Suggestions about MetS by Chinese diabetes society. Chin J Diabetes.

[18] Chinese Society of Geriatric Medicine, Hypertension Branch; Beijing Hypertension Prevention and Treatment Association; National Clinical Research Center for Geriatric Diseases Chinese Guidelines for the Management of Hypertension in the Elderly. Chinese Journal of Hypertension. 2023;31(6): 508–538.

[19] Zheng J, Hu Y, Xu H, Lei Y, Zhang J, Zheng Q (2023). Normal-weight visceral obesity promotes a higher 10-year atherosclerotic cardiovascular disease risk in patients with type 2 diabetes mellitus-a multicenter study in China. Cardiovasc Diabetol.

[20] Durrleman S, Simon R (1989). Flexible regression models with cubic splines. Statist Med.

[21] Welzel TM, Graubard BI, Zeuzem S, El-Serag HB, Davila JA, McGlynn KA (2011). MetS increases the risk of primary liver cancer in the United States: a study in the SEER-Medicare database. Hepatology.

[22] Stepanova M, Rafifiq N, Younossi ZM (2010). Components of MetS are independent predictors of mortality in patients with chronic liver disease:a population-based study. Gut.

[23] Kanwal F, Kramer JR, Li L, Dai J, Natarajan Y, Yu X (2020). Effect of metabolic traits on the risk of cirrhosis and hepatocellular cancer in nonalcoholic fatty liver disease. Hepatology.

[24] Irene P, Gianluca SB (2019). Nonalcoholic fatty liver disease: basic pathogenetic mechanisms in the progression from NAFLD to NASH. Transplantation.

[25] Elizabeth EP, Vincent WW, Mary R (2021). Non-alcoholic fatty liver disease. Lancet.

[26] Bronwyn AK, Chapman MJ, Anatol K, Norman EM (2014). HDL-targeted therapies: progress, failures and future. Nat Rev Drug Discov.

[27] Joerg H, Ludger S (2021). Metabolic-associated fatty liver disease and lipoprotein metabolism. Mol Metab.

[28] Bajaj JS, Nagy LE (2022). Natural history of alcohol-associated liver disease: understanding the changing landscape of pathophysiology and patient care. Gastroenterology.

[29] O’Shea RS, Dasarathy S, McCullough AJ (2010). Practice guideline committee of the american association for the study of liver diseases; practice parameters committee of the american college of gastroenterology. Alcoholic Liver Dis Hepatol.

[30] Neil DS, Meritxell VC, Juan GA, Mohamed A, Ahmad A, Josepmaria A (2019). Alcohol-related liver disease is rarely detected at early stages compared with liver diseases of other etiologies worldwide. Clin Gastroenterol Hepatol.

[31] Bataller R, Arab JP, Shah VH (2022). Alcohol-associated hepatitis. N Engl J Med.

[32] Ruhl CE, Everhart JE (2005). Joint effects of body weight and alcohol on elevated serum alanine aminotransferase in the United States population. Clin Gastroenterol Hepatol.

[33] Zhe S, Youming L, Chaohui Y, Yi S, Lei X, Chengfu X (2010). A cohort study of the effect of alcohol consumption and obesity on serum liver enzyme levels. Eur J Gastroenterol Hepatol.

[34] Vincent M, Lucia P, Alessandro M, Juan P, Carmen NJ, Mehdi S (2022). Burden of liver disease progression in hospitalized patients with type 2 diabetes mellitus. J Hepatol.

[35] Claire D, Pierre B, Alexandre L, Flavien D, Line CW, Guillaume L (2020). A model to identify heavy drinkers at high risk for liver disease progression. Clin Gastroenterol Hepatol.

[36] Lieber CS (1975). Alcohol and malnutrition in the pathogenesis of liver disease. JAMA.

[37] Michel L, Nadja CS, Vilhelm AB (2011). Metabolism, genomics, and DNA repair in the mouse aging liver. Curr Gerontol Geriatr Res.

[38] James MW, Fiona MW (2018). Diverse mechanisms for endogenous regeneration and repair in mammalian organs. Nature.

[39] Yee HY, Yixuan Z, Juan PA, Wenjing N, Xiaoming X, Junping S (2023). Alcohol intake thresholds among individuals with Steatotic liver disease. JAMA Netw Open.

